# The Unstimulated Salivary Flow Rate in a Jordanian Healthy Adult Population

**DOI:** 10.4021/jocmr2009.10.1267

**Published:** 2009-10-16

**Authors:** Faleh A. Sawair, Soukaina Ryalat, Mohammad Shayyab, Takashi Saku

**Affiliations:** a Department of Oral and Maxillofacial Surgery, Oral Medicine, Oral pathology and Periodontology, Faculty of Dentistry, University of Jordan. Amman, Jordan.; b Division of Oral Pathology, Department of Tissue Regeneration and Reconstruction, Niigata University Graduate School of Medical and Dental Sciences, Niigata, Japan.

## Abstract

**Background:**

Early diagnosis of xerostomia is very important for oral health. The purpose of this study was to determine the unstimulated whole salivary flow rates (UWSFR) in a Jordanian Arab population aged 15 years and older. The effect of age, gender, height, weight, body mass index (BMI), smoking, alcohol consumption, and dental conditions, on UWSFR was also investigated.

**Methods:**

The study was conducted on 244 subjects, 110 males and 134 females, with an average age of 33 ± 15.5 years. They were healthy, unmedicated, and with no history of dry mouth. Unstimulated whole saliva was collected during five minutes, and UWSFRs (ml/min) were determined. Data were analyzed by univariate analysis and multivariate regression analysis.

**Results:**

The mean UWSFR was 0.46 ± 0.25 ml/min (range: 0.10-1.6 ml/min). Eighteen patients (7.4%) had UWSFR between < 0.20 ml/min. In univariate analysis, UWSFR was significantly affected by age, BMI, number of missing and restored teeth, and DMFT score. Regression analysis revealed that only age and number of missing teeth were of significance in explaining the variability of the UWSFR.

**Conclusions:**

We established basic standard values of UWSFR to be used in the evaluation of Jordanian patients with complaints of xerostomia and to be compared to data reported in other studies. UWSFR 0.1 ml/min could be considered the cut-off value that distinguishes normal from abnormal salivary function in this healthy unmedicated population.

**Keywords:**

Whole saliva flow rate; Unstimulated; Jordan

## Introduction

The knowledge of normal salivary flow rate (SFR) is extremely important when treating dental patients. Early diagnosis and treatment of hyposalivation will preserve the health of oral structures and lower the incidence of dental caries, fungal infections, and other oral diseases that could result from insufficient SFR [1,2]. Xerostomia can be caused by many reasons including medications, head and neck irradiation, anxiety or depression, Sjogren's syndrome, and systemic diseases such as diabetes mellitus and some autoimmune disorders [1]. Although many methods can be used to evaluate salivary function, collecting unstimulated whole saliva is the method most frequently used. This method can be easily conducted without any special equipment or physical stress on subjects [3].

Several studies were conducted to establish a critical limit of UWSFR to separate subjects with salivary gland hypofunction from those with normal gland function [4-8]. No consensus has been reached; the critical limit was 0.1 ml/min by some researchers [4,5], while by others it was 0.15 ml/min [6], 0.16 ml/min [7], or 0.20 ml/min [8]. The normal ranges for SFR were also variable (Table 1).

**Table 1:** Results of previous studies that measured unstimulated whole salivary flow rate (UWSFR)

Authors	Country	Number of subjects,	Age	UWSFR ml/min	Notes
		(male/female)		(mean ± SD)	
Yamamoto et al [3]	Japan	200, (100/100)	22 - 29	0.053 ± 0.032	H/UM
Fenoll-Palomares et al [6]	Spain	159, (52/107)	44 ± 14	Range: 0.10-2.0, median: 0.48	H/UM
Flink et al [9]	Sweden	1420, (663/757)	≥ 20	0.29 ± 0.24	Dry/M
Bergdahl [10]	Sweden	1427, (669/758)	20 - 69	0.33 ± 0.26 (male)	Dry/M
				0.26 ± 0.21 (female)	
Percival et al [11]	UK	116, (55/61)	≥ 20	0.50 ± 0.04 (male) (mean ± SEM)	H/UM
				0.33 ± 0.03 (female) (mean ± SEM)	
Shern et al [12]	USA	51, (25/26)	54 ± 19	Mean: 0.61	H/UM
Marton et al [13]	Hungary	24, (?)	67 ± 8	0.39 ± 0.25	CD
					

SD: standard deviation, H/UM: healthy and unmedicated. CD: complete dentures, Dry/M: patients who had oral dryness or other oral complaints or those who take xerostomic drugs were not excluded, SEM: standard error of mean.

However, it is difficult to compare SFR reported by different studies due to variations in study design. Differences exist in terms of sample size, patients gender, age, height, weight, BMI, salivary gland size, diet, or bite force [3, 6, 9, 10, 12-14] and in many of these studies these factors was not studied in multivariate analysis. Many investigators studied the prevalence of hyposalivation and, therefore, their samples included patients with systemic diseases or those taking xerostomic medications or had complaints of oral dryness [9,10]. Furthermore, the results of different studies are difficult to compare due to variations in circumstances under which UWSFR was collected. Saliva is secreted in a circadian and circannual rhythm and the time of measurement of UWSFR strongly influences SFR [15]. Factors such as thinking of food, visual stimulation, body posture, degree of room lighting, nausea, earlier chewing of gum or physical exercise, could influence SFR and are difficult to standardise in different studies [16].

Bjornstad and Crossner [17] have conducted a comparative study and found significant variations in SFRs between subjects from Greenland and Sweden and highlighted the importance of being cautious when exchanging reference data between people from different cultures or ethnic groups. This variation may affect the critical limit of SFR that separates patients with xerostomia from those with normal flow rates. Significant differences in UWSFR were also reported when children from USA and Brazil were compared [18]. To the best of our knowledge, no studies were conducted in Jordan or in the Arab world to establish normal ranges of SFR.

The aim of this study was to evaluate the normal UWSFR and identify independent factors affecting this flow among healthy adult Jordanian population. The results may help in establishing the critical limit of UWSFR that separates Jordanian subjects with normal salivary gland function from those with gland hypofunction and for comparison with data reported in other studies.

## Materials and Methods

The salivary glands are fully developed in terms of flow rate at the age of 15 years [19]. Therefore, this study was conducted on 244 volunteers ranging in age from 15 to 76 years chosen in an at-random way from subjects who attended the Department of Dentistry, University of Jordan Hospital, Amman, Jordan, for dental treatment in the period between January and April 2009. The purpose of the study was explained and all subjects had given their informed consent for participation. Patients were medically healthy without taking any medications known to affect salivary flow. They were excluded if they had acute or chronic diseases of the oral mucosa or salivary glands including xerostomia or oral burning sensation, if they had moderate or severe periodontal disease, or if they had partial or complete dentures or orthodontic appliances. Information about age, gender, height, weight, BMI, smoking history, and alcohol consumption were collected. BMI was recorded as: underweight <18.5 kg/m^2^; normal weight 18.5-24.9 kg/m^2^; over weight 25-29.9 kg/m^2^; and obese ≥ 30 kg/m^2^.

Patients were then clinically examined to rule out acute or chronic diseases of oral mucosa or salivary glands. DMFT (decayed, missing, and filled teeth) index of all permanent teeth excluding third molars was determined for each subject under artificial light, using a dental explorer, flat-surface mouth mirror, gauze, and compressed air according to the WHO diagnostic criteria [20]. Under standard temperature and humidity conditions, UWSFRs were determined in the morning between 10 a.m. and noon. Amounts of liquids ingested by subjects were not studied but all subjects refrained from smoking or intake of any food or beverage at least one hour before saliva collection [21]. Subjects were instructed to relax for 5 minutes and to swallow all saliva present in their mouths before starting saliva collection. While seated and leaning forward, they were told to spit all the saliva they produce into a graduated test tube through a glass funnel. The unstimulated whole saliva collected for 5 minutes was then measured by volume and expressed as millilitres per minute (ml/min) [21]. Clinical examinations and SFR measurements were made by one examiner (S.R.).

Statistical analysis was performed using SPSS for Windows release 16.0 (SPSS Inc., Chicago, IL, USA). Descriptive statistics were generated. Student's t-test, One-Way-ANOVA test, and Pearsons Correlations test (r) were used to examine differences between groups. When One-Way-ANOVA test was conducted, post hoc multiple comparisons were used to see which pairs of means were statistically significant. Multiple Stepwise Linear Regression analysis was applied for the determination of the best predictors of UWSFR among gender, age, height, weight, BMI, smoking and alcohol consumption, and dental variables. Coefficients of regression and 95% Confidence Intervals (CI) were calculated for each significant independent variable. Results were considered significant if P-values were less than 0.05.

## Results

### Subjects

Characteristics of the subjects recruited in the present study are shown in Table 2. They were 244 in number, 110 males and 134 females, and their age ranged from 15 to 76 years with an average of 33 years (± 15.5 years). Mean age for males was 36.0 ± 15.9 years and that for females was 30.5 ± 14.9 years (P = 0.006). Smokers were significantly older than non-smokers (36.8 ± 16.5 vs. 31.5 ± 15.0 years, P = 0.02). There was statistically significant positive correlation between age and BMI (r = 0.53, P < 0.001). Mean BMI for males was significantly higher than that for females (24.9 ± 3.9 vs. 22.6 ± 4.3, P < 0.001). A significant positive correlation was found between age and number of decayed teeth (r = 0.38, P < 0.001), number of missing teeth (r = 0.50, P < 0.001), number of restored teeth (r = 0.34, P < 0.001), and DMFT score (r = 0.63, P < 0.001). Males and females did not differ significantly in the mean number of decayed, missing, and restored teeth, or in DMFT score.

**Table 2:** Patients characteristics and its relation to unstimulated whole salivary flow rate (UWSFR)

Variables		Number (%)	UWSFR (ml/min)		
			Mean (SD)	Range	P value*
**Gender**	Male	110 (45.1)	0.44 (0.24)	0.10 - 1.30	0.16
	Female	134 (54.9)	0.48 (0.26)	0.12 - 1.60	
					
**Age (year)**	15 - 19	32 (13.1)	0.71 (0.32)	0.20 - 1.30	< 0.001
	20 - 29	106 (43.4)	0.47 (0.25)	0.12 - 1.60	
	30 - 39	32 (13.1)	0.41 (0.17)	0.20 - 0.82	
	40 - 49	28 (11.5)	0.41 (0.17)	0.10 - 0.72	
	50 - 59	29 (11.9)	0.37 (0.15)	0.18 - 0.78	
	≥ 60	17 (7.0)	0.30 (0.15)	0.12 - 0.50	
					
**Smoking**	Non-smoker	175 (71.7)	0.47 (0.25)	0.12 - 1.60	0.45
	Smoker	69 (28.3)	0.44 (0.25)	0.10 - 1.24	
					
**Alcohol**	No	238 (97.5)	0.47 (0.25)	1.00 - 1.60	0.20
	Yes	6 (2.5)	0.33 (0.12)	0.20 - 0.50	
					
**Height (m)**	< 1.6	20 (8.2)	0.49 (0.33)	0.20 - 1.60	0.25
	1.6 - 1.69	95 (38.9)	0.47 (0.24)	0.12 - 1.24	
	1.7 - 1.79	73 (29.9)	0.44 (0.22)	0.12 - 1.10	
	≥ 1.8	31 (12.7)	0.54 (0.30)	0.10 - 1.30	
	Missing data	25 (10.2)			
					
**Weight (kg)**	< 60	74 (30.3)	0.51 (0.27)	0.12 - 1.60	0.10
	60 - 79	87 (35.7)	0.47 (0.26)	0.12 - 1.30	
	≥ 80	59 (24.2)	0.42 (0.21)	0.10 - 1.00	
	Missing data	24 (9.8)			
					
**BMI¶**	Underweight	12 (4.9)	0.59 (0.39)	0.20 - 1.60	0.007
	Normal weight	134 (54.9)	0.50 (0.28)	0.12 - 1.30	
	Overweight	53 (21.7)	0.41 (0.22)	0.10 - 1.24	
	Obese	20 (8.2)	0.35 (0.14)	0.12 - 0.60	
	Missing data	25 (10.2)			

*P value of Student’s t test or ANOVA, BMI¶: underweight < 18.5 kg/m^2^, normal weight 18.5-24.9 kg/m^2^, overweight 25-29.9 kg/m^2^, obese ≥ 30 kg/m^2^.

### Salivary flow rate

The mean UWSFR was 0.46 ± 0.25 ml/min and the median flow rate was 0.42 ml/min (range 0.10-1.6 ml/min, percentile 5 = 0.16 ml/min and percentile 95 = 0.92 ml/min). None of the patients had UWSFR less than 0.1 ml/min. However, 18 patients (7.4%) had UWSFR < 0.20 ml/min.

### Relationship between UWSFR and patients variables

The relationships between UWSFR and patients variables are shown in Table 2. Mean UWSFR in males, smokers or alcohol drinkers did not differ significantly from that in females, non-smokers or non-alcohol drinkers. No significant correlation was found between UWSFR and patients height (r = 0.06, P = 0.36) or weight (r = -0.12, P = 0.08), the differences in UWSFR were not significant when the subjects were separated into four groups according to their height or into three groups according to their weights.

UWSFRs decreased significantly as age increased (r = -0.36, P < 0.001). Post hoc multiple comparisons revealed that UWSFR in subjects younger than 20 years old was significantly higher than the flow rate detected in the other age groups, UWSFR in subjects 20-29 years old was significantly higher than those ≥ 60 years old, but the differences in flow rates between patients in the age groups 30-39, 40-49, 50-59 were not statistically significant. Another significant negative correlation was also found between UWSFR and BMI (r = -0.18, P = 0.007). The major and statistically significant difference revealed by post hoc comparisons was between obese subjects and those who were underweight or of normal weight; differences between the other groups were not statistically significant (Table 2).

Patients' dental conditions are shown in Table 3. Significant negative correlations were found between UWSFR and the number of missing teeth (r = -0.29, P < 0.001), number of restored teeth (r = -0.18, P = 0.005), and DMFT score (r = -0.31, P < 0.001).

**Table 3:** Patients dental conditions and its relation to unstimulated whole salivary flow rate (UWSFR)

Variables		Number (%)	UWSFR (ml/min)		
			Mean (SD)	Range	P value*
Decayed teeth	None	66 (27.0)	0.50 (0.31)	0.12 - 1.30	0.28
	1 - 5	136 (55.7)	0.46 (0.24)	0.10 - 1.60	
	> 5	42 (17.2)	0.42 (0.15)	0.16 - 0.70	
					
Missing teeth	None	137 (56.1)	0.54 (0.28)	0.12 - 1.60	< 0.001
	1 - 5	94 (38.5)	0.38 (0.15)	0.10 - 0.78	
	> 5	13 (5.3)	0.24 (0.08)	0.12 - 0.40	
					
Restored teeth	None	56 (23.0)	0.57 (0.27)	0.12 - 1.30	0.002
	1 - 5	143 (58.6)	0.43 (0.23)	0.10 - 1.24	
	> 5	45 (18.4)	0.42 (0.25)	0.12 - 1.60	
					
DMFT	0	18 (7.4)	0.64 (0.33)	0.16 - 1.30	< 0.001
	1 - 5	83 (34.0)	0.50 (0.27)	0.12 - 1.24	
	6 - 10	73 (29.9)	0.46 (0.25)	0.10 - 1.60	
	11 - 15	48 (19.7)	0.41 (0.14)	0.16 - 0.70	
	> 15	22 (9.0)	0.29 (0.13)	0.12 - 0.70	

(*P value of ANOVA)

The differences in flow rates between patients subgroups according to number of missing teeth, filled teeth, or DMFT score were statistically significant (Table 3). All post hoc comparisons between the three groups of subjects according to number of missing teeth (none, 1-5, and >5 teeth missing) were statistically significant. In terms of restored teeth, patients who had no restored teeth had significantly higher flow rate compared with patients who had 1-5 or more filled teeth, differences between patients who had 1-5 and those who had > 5 filled teeth were not statistically significant. Post hoc comparisons revealed that UWSFR in subjects who had zero DMFT score was significantly higher than that detected in all groups having DMFT score > 5. UWSFR in subjects who had > 15 DMFT score was significantly lower than that detected in all groups having DMFT score < 10. The correlation of UWSFR with number of decayed teeth did not reach statistical significance (r = -0.12, P = 0.06) and differences in flow rates between the three groups of patients according to number of decayed teeth were not statistically significant.

### Multivariate analysis

Multivariate regression analysis revealed two factors as significant independent predictors of UWSFR (Table 4). When the effects of other factors were controlled, age and number of missing teeth were found to be the most important factors. For every one year increase in age, resting salivary flow rate would decrease 0.002 to 0.006 ml/min (average 0.004 ml/min, P < 0.001). In addition, for every one tooth loss, UWSFR would decrease by 0.002 to 0.027 ml/min (average 0.015 ml/min, P = 0.021).

**Table 4:** Significant independent predictors of unstimulated whole salivary flow rate (UWSFR) in multivariate analysis

	B	β	95% CI (B)	P value
**Constant**	0.615	-	0.544 to 0.687	< 0.001
**Age**	-0.004	-0.250	- 0.006 to - 0.002	< 0.001
**Missing teeth**	-0.015	-0.162	- 0.027 to - 0.002	0.021

B: un-standardized regression coefficient. β: standardized regression coefficient, CI: confidence interval. R = 36%, adjusted R^2^ = 12%

## Discussion

As mentioned before, there is no general agreement about UWSFR that distinguishes normal patients from those with hyposalivation; the value has ranged between 0.1 and 0.2 ml/min [4-8]. As a result, many investigators classify their patients into three groups: those with very low SFR (UWSFR < 0.1 ml/min); those with low SFR (UWSFR = 0.1-0.19 ml/min); and those who have normal SFR (UWSFR ≥ 0.2 ml/min) [9]. In this study, considerable variation was observed in UWSFR, 0.1 to 1.6 ml/min and 90% of the subjects (those between percentile 5 and 95) had UWSFR that ranged between 0.16 and 0.92 ml/min. No patients had flow rate below 0.1 ml/min but 18 patients (7.4%) had UWSFR between 0.10 and 0.19 ml/min. Although the latter 7.4% of the patients are considered having low UWSFR according to the previous classification, none of them had signs or symptoms of oral dryness. This indicates high inter-individual variability in the amount of saliva necessary for oral health. Since 0.1 ml/min UWSFR was the lowest rate of secretion found in this study and because patients who had complaints of oral dryness were excluded, 0.1 ml/min UWSFR could be considered the cut-off value that distinguishes normal from abnormal salivary function in this healthy unmedicated population.

Considerable variation in mean UWSFR in adult patients is evident when studies carried out in different countries are compared. The mean UWSFR ranged between 0.053 ml/min [3] to 0.61 ml/min [12]. Although comparisons are difficult to be made between studies due to considerable variations in study designs, the mean UWSFR for Jordanian subjects measured in this study is nearly comparable to that reported by studies conducted in Spain [6] and UK [11], higher than that reported in a Japanese study [3], but lower than that reported in a study conducted in USA [12] on healthy unmedicated adult patients.

For accurate evaluation of main UWSFR, it is important to control for the significant independent factors that influence SFR using multivariate statistical analysis. In this study, only age and number of missing teeth were independent predictors of UWSFR. Therefore, these two factors should be controlled when comparisons are made between Jordanian patients. In adult healthy unmedicated Jordanians, UWSFR decreased significantly as age and number of missing teeth increased. However, in the multivariate regression model, these two independent variables explained only 12% (R2) of the differences in SFRs between individuals of the study sample. The low prediction rate highlights the need for additional studies to explain the variations in SFRs in this population.

Unstimulated salivary flow is produced primarily by the submandibular salivary glands (65-70%), with the parotid and sublingual glands providing 20% and 7% to the flow, respectively [16]. Research has shown diminution of both stimulated and unstimulated submandibular SFR with increasing age [22] but the reasons behind this finding were not clear. In this study, none of the patients suffered from systemic or salivary gland diseases and none were taking medication that could compromise SFR. Therefore, our finding that UWSFR decreases significantly with increasing age, a finding in agreement with several previous reports [6,7,11,23], is difficult to explain. The reduction could result from structural changes affecting the submandibular salivary glands. In fact, age-related gradual loss of secretory tissue of the submandibular glands and replacement with fat and connective tissue has been reported [24]. In contrast to submandibular glands, in healthy individuals parotid gland function is not affected by the aging process as was shown by some investigators [11,25,26] and, since the parotid glands supply about 60% of the stimulated whole saliva [16], reports have shown no decrease in stimulated whole salivary flow rates with increasing age [27,28]. In fact, some reports have shown an unexplainable increase in stimulated salivary gland secretion with increasing age [7,12].

The effect of the number of teeth on UWSFR has not been fully investigated. The results in this study showed that UWSFR decreased significantly as number of missing teeth increased. Flink et al [9] have reported that patients who had fewer than 20 teeth had significantly more prevalent very low stimulated (< 0.70 ml/min) and unstimulated (< 0.10 ml/min) SFRs. The authors explained this relationship by the possible increase in caries activity and subsequent loss of teeth secondary to reduced salivary flow. In this study no significant association was found between the number of decayed teeth and UWSFR. Since the relationship between caries and SFR was only confirmed in patients with very low (< 0.10 ml/min) SFR [29], this finding was expected since we did not recruit subjects who had suspected hyposalivation. An alternative explanation to the relation between missing teeth and UWSFR was shown by Yeh et al [23] who have reported a significant correlation between salivary flow rate and maximum bite force independent of age and gender, a decrease in bite force strength was associated with a decrease in UWSFR. According to the authors, the loss of teeth was considered an apparent reason responsible for the decrease in bite force and subsequent decrease in salivary flow rate. Therefore, it is unclear whether hyposalivation leads to loss of teeth or loss of teeth decreases the SFR, and further studies to explore this topic are needed.

Conflicting results were reported on UWSFR in relation to gender. Our data indicated no significant difference between males and females in terms of resting salivary flow. Similar findings were reported by Shern et al [12]. In contrast, some investigators have found that males had higher secretion rates of unstimulated saliva compared with females [9,11]. It is possible that the effect of gender is not genuine and secondary to other factors highlighting the importance of multivariate analysis. Some investigators have attributed this difference to greater glandular mass in males [30], differences in hormonal patterns [31], or differences in BMI [3]. Therefore, when the effects of body profiles and glandular size, two correlated factors [32], were taken into account, the significant gender difference disappeared [30]. The significant correlation of UWSFR with BMI in the present study was in agreement with previous reports [3,11]. Since the correlation coefficient between BMI and age was moderately strong in this sample, the result that BMI lost its significant effect on UWSFR was unsurprising result in multivariate analysis. In agreement with earlier reports [6], we found no differences in UWSFR existing between smokers and non-smokers.

We conclude that UWSFR in this adult Jordanian population ranged between 0.1 to 1.6 ml/min and patients with as low as 0.10 ml/min had no signs or symptoms of oral dryness. To evaluate our results about the lowest normal rate of UWSFR, it would be necessary further to study patients diagnosed with xerostomia. Of the demographic factors/parameters we tested in this study, only age and number of missing teeth contributed consistently to the variability in resting salivary flow rate in this population.
